# New mechanism of LncRNA: In addition to act as a ceRNA

**DOI:** 10.1016/j.ncrna.2024.06.002

**Published:** 2024-06-03

**Authors:** Jiahao Zhang, Huike Zhu, Linjing Li, Yuting Gao, Boyi Yu, Guorong Ma, Xiaodong Jin, Yingbiao Sun

**Affiliations:** a Department of Toxicology, School of Public Health, Lanzhou University, Lanzhou, 730000, China; b Institute of Modern Physics, Chinese Academy of Sciences, Lanzhou 730000, China; c University of Chinese Academy of Sciences, Beijing, 100049, China; d College of Life Sciences, Northwest Normal University, Gansu Province, Lanzhou, 730070, China; e The First Clinical Medical College of Gansu University of Chinese Medicine Gansu Provincial Hospital, Lanzhou, 730000, China

**Keywords:** LncRNA, Epigenetic, Post-translational, Scaffolding protein, Peptide

## Abstract

Long non-coding RNAs (LncRNAs) are a class of RNA molecules with nucleic acid lengths ranging from 200 bp to 100 kb that cannot code for proteins, which are diverse and widely expressed in both animals and plants. Scholars have found that lncRNAs can regulate human physiological processes at the gene and protein levels, mainly through the regulation of epigenetic, transcriptional and post-transcriptional levels of genes and proteins, as well as in the immune response by regulating the expression of immune cells and inflammatory factors, and thus participate in the occurrence and development of a variety of diseases. From the downstream targets of lncRNAs, we summarize the new research progress of lncRNA mechanisms other than miRNA sponges in recent years, aiming to provide new ideas and directions for the study of lncRNA mechanisms.

## Graphical abstract

**Figure:**
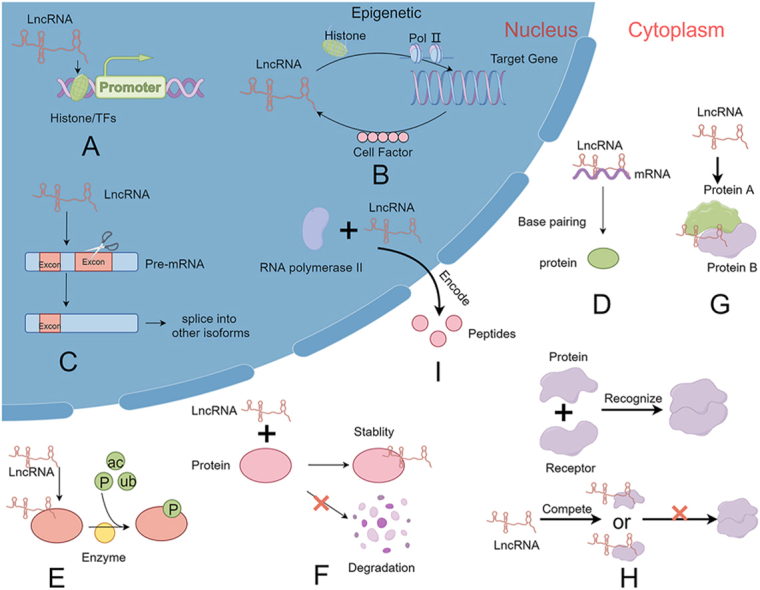

## Abbreviations

AR: Androgen receptor
AS: Alternative splicing
CeRNA: Competing endogenous RNA
CircRNA: Circular RNA
COPI: COATOMER protein complex I
CRC: Colorectal cancer
CVD: Cardiovascular disease
DNMT: DNA methyltransferase
GBM: Glioblastoma multiforme
GC: Gastric cancer
HATs: Histone acetyltransferases
HDAC1: Histone deacetylase 1
HnRNP: Heterogeneous nuclear ribonucleoprotein
HnRNPU: Heterogeneous nuclear ribonuclear protein U
IRF3: Interferon regulatory factor 3
LAD: Lung adenocarcinoma
LncRNA: Long non-coding RNA
MiRNA: Micro RNA
NcRNA: Non-coding RNA
NSCLC: Non-small cell lung cancer
SMC: Smooth muscle cells
SR: Serine/arginine-rich splicing factor
SRF: Serum response factor
SUZ12: Zeste 12 repressor
TFs: Transcription factors
TNBC: Triple-negative breast cancer
VLDL: Very low-density lipoprotein
YBX1: Y-Box binding protein 1

## 1 Introduction

Non-coding RNAs (NcRNAs) are a class of RNAs that do not encode proteins during transcription, have recently been recognized as an important regulatory factor. NcRNAs can be divided into lncRNAs, micro RNAs (miRNAs) and circular RNAs (circRNAs) [1]. LncRNAs are ncRNAs of more than 200 nt, that do not translate proteins, but their regulatory role in mediating cell processes cannot be ignored [2]. It is widely involved in the development and treatment of diseases, such as tumors, cardiovascular diseases（CVD）, diabetes, and arthritis, etc [[3], [4], [5], [6], [7], [8]].

The relationship between lncRNAs and disease is complex, especially in oncology research. One lncRNA can regulate the progression of multiple tumors. LncRNA HOXA-AS2 has been shown to be associated with the progression of breast, gastric, gallbladder, hepatocellular, and pancreatic cancers [9]. LncRNA AFAP1-AS1 has also been shown to be involved in the onset and progression of various malignant tumors [10]. As well, a particular tumor can be regulated by multiple lncRNAs. 61 lncRNAs have been found to be differentially expressed in the prostate alone, 68 lncRNAs have been found to be associated with survival and risk of developing gastric cancer (GC), and 74 lncRNAs are involved in the construction of the ceRNA network in hepatocellular carcinoma [[11], [12], [13]]. It is the complex relationship between lncRNAs and disease that has led to the promise of lncRNAs as potential biomarkers of disease, and therefore it has become important to study the mechanism of action of lncRNAs.

Mechanism research of LncRNA originated from the discovery of competing endogenous RNA(ceRNA). CeRNA is not a specific type of RNA, but a mechanism of interaction between RNAs, which can act as a sponge for miRNAs and attenuate the inhibitory effect of miRNAs on mRNAs, mainly including pseudogenes, lncRNAs, and circRNAs [[14], [15], [16]]. The hypothesis of ceRNAs was first presented at CELL in 2011 by Paolo Pandolfi, who proposed through his findings and extrapolated hypotheses that all RNAs can communicate with each other through miRNAs [17]. The ceRNA hypothesis has attracted the attention of a wide range of scholars since it was proposed, and Linc-MD1 was the first ceRNA that was shown to be able to participate in the physiological functions of humans and mice [18].

The regulatory mechanism of lncRNAs as ceRNAs is now widely recognized, but there is still much uncertainty about the mechanism of action between lncRNAs and miRNAs, as well as the existence of other forms of regulatory mechanisms. Since ceRNAs are more abundantly studied, the involvement of lncRNAs in human physiological and pathological processes through other forms deserves further research.

## 2 LncRNA regulation at the transcriptional level

### 2.1 Directly regulates gene transcription

**Fig. 1:**
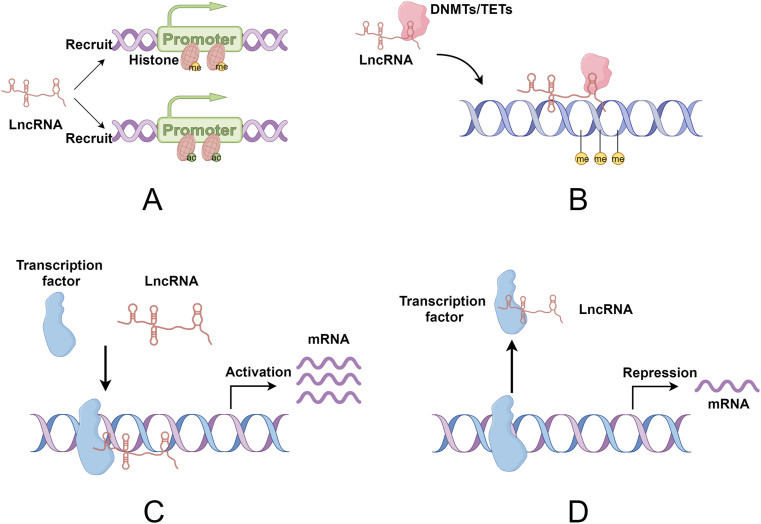
LncRNAs regulate gene transcription through epigenetic or TFs. By Figdraw. **(A)** LncRNAs are involved in histone modification and are capable of recruiting histones with different functions to the promoter regions of target genes to promote or repress their transcription. **(B)** LncRNAs promote (or inhibit) DNA methylation of target genes with the help of DNMTs (or TETs). **(C)** LncRNAs recognize TFs and facilitates the binding of TFs to target genes to promote their transcription. **(D)** LncRNAs occupy the binding sites of TFs and target genes, inhibiting the binding of TFs to target genes and suppressing the transcription of them.

LncRNA, as a novel epigenetic factor, is able to bind to the promoter region of genes. Through DNA methylation, chromatin remodeling, histone modification, etc., the transcription level of DNA will be enhanced or reduced although the DNA sequence is not changed [[19], [20], [21]]. Additionally, lncRNAs can interact with target genes by binding to various transcription factors (TFs) to promote or repress the transcription of target genes [22]. LncRNAs are based on epigenetic or with the help of TFs to accomplish the information communication with mRNAs and realize the regulation of target genes at the transcriptional level (Fig. 1).

#### 2.1.1 LncRNA regulates target gene transcription through histone modification

Histones are one of the most important bridges connecting communication between lncRNA and mRNA which are mainly found in the nucleus. Histones are able to chemically modify DNA through modifications (such as methylation and acetylation), thus affecting the expression of certain genes [23]. The involvement of lncRNAs in the process of histone methylation or acetylation can be observed (Fig. 1A). Lung adenocarcinoma (LAD) is the most common type of non-small cell lung cancer (NSCLC) with a high degree of malignancy [24]. Chemotherapy is one of the main modalities of cancer treatment, however, drug resistance developed during chemotherapy remains an unavoidable challenge in cancer treatment [25]. LncRNA MARCKSL1-2 was found to attenuate docetaxel resistance in LAD cells by recruiting the zeste 12 repressor (SUZ12) to the histone deacetylase 1 (HDAC1) promoter to strengthen histone H3 lysine 27 methylation of HDAC1 promoter, and thus reduce HDAC1 expression. Meanwhile the reduction of HDAC1 expression upregulated miR-200b expression by attenuating the inhibitory effect on histone acetylation modification of the miR-200b promoter, thereby attenuating docetaxel resistance in LAD cells [26]. Histone acetylation is also a type of histone modification. LncRNA MALAT1 is increased in non-obese diabetic mice compared to wild-type mice. Further research confirmed that lncRNA MALAT1 inhibits PDX-1 expression by reducing H3 histone acetylation of the PDX-1 promoter, thereby inhibiting insulin secretion [27].

In histone modification, lncRNAs, in addition to mediating histone activity, can also enhance or inhibit the transcription of target genes by recruiting histones with different functions to the promoter regions of target genes (Fig. 1A). Unlike lncRNAs, which are directly involved in the regulation of histone activity, these lncRNAs only serve as a scaffold connecting histones to target genes and are not involved in histone methylation or acetylation processes. OBSCN-AS1 is a lncRNA originating from the negative strand of the OBSCN gene, and the cytoskeletal protein obscurin encoded by OBSCN is closely associated with triple-negative breast cancer (TNBC） development. Further experiments showed that up-regulation of lncRNA OBSCN-AS1 expression resulted in a significant increase in RNA polymerase II occupancy at the lncRNA OBSCN-AS1 promoter and enrichment of histone H3K4me3, which promotes gene transcription, at the promoter, whereas down-regulation of lncRNA OBSCN-AS1 decreased RNA polymerase II occupancy and enriched histone H3K9me3, which inhibits gene transcription. Activation of lncRNA OBSCN-AS1 transcription by CRISPR technology indirectly promotes the transcription and translation of OBSCN-encoded proteins. Thereby, the invasive and metastatic ability of TNBC cells was reduced, and lncRNA OBSCN-AS1 is expected to be a therapeutic target for TNBC [28].

#### 2.1.2 LncRNA regulates target gene transcription through DNA methylation

DNA methylation is the binding of a methyl group to a target gene by the action of DNA methyltransferase (DNMT) without altering the DNA sequence [29]. Such modifications affect not only the structure and function of DNA, but also the expression and function of genes. LncRNAs bind DNMTs or DNA demethylases (e.g., the TET family) to target genes and affect the DNA methylation of target genes (Fig. 1B). DNA methylation can promote gene expression. Systemic lupus erythematosus is a complex autoimmune disease with unclear mechanisms and high heritability [30]. Several studies have shown that the aberrant expression of IRF8 is closely related to the development of systemic lupus erythematosus. There exists a SNP site rs2280381 downstream of IRF8 that specifically enhances IRF8 expression. This site binds to the promoter region of IRF8 and forms a chromatin loop with the help of lncRNA AC092723.1, while AC092723.1 recruits the demethylase TET1 into the IRF8 promoter and represses IRF8 transcription by decreasing the methylation level of the IRF8 promoter, leading to the dysregulation of IRF8 [31].

DNA methylation can also inhibit gene expression. Tamoxifen resistance is one of the clinical challenges in hormone receptor-positive breast cancer and dysregulation in autophagy function have been proved to be a potential mechanism for the development of tamoxifen resistance [32,33]. Beclin1, as an autophagy-related gene, its expression was positively correlated with autophagy level, and DNMT3B-mediated methylation of the Beclin1 promoter region could effectively inhibit the expression of Beclin1. It was demonstrated that LncRNA H19 attenuates the interaction of DNMT3B with the Beclin1 promoter region by binding to and inhibiting s -adenosine-type homocysteine hydrolase, which promotes Beclin1 expression and enhances autophagic activity [34].

#### 2.1.3 LncRNA regulates target gene transcription through transcription factors

TFs are specialized proteins that bind to specific DNA sequences and activate or repress gene transcription. TFs-mediated epigenetic modifications are capable of regulating gene expression in disease occurrence and progression [35]. LncRNAs are able to recruit TFs to target genes and activate their transcriptional activity, thus indirectly promoting gene transcription and expression (Fig. 1C). Disorders of intestinal smooth muscle contraction underlie many gastrointestinal disorders [36]. Carmn is a lncRNA that is highly expressed in smooth muscle cells（SMC）of the human and mouse gastrointestinal tracts and can affect smooth muscle contraction and trigger human visceral myopathy in the gastrointestinal tract. Deletion of lncRNA Carmn represses the expression of a variety of SMC contraction genes, including the MYLK gene. Serum response factor (SRF) is a highly conserved transcription factor that plays an important role in cell proliferation, apoptosis, and immune responses. It was found that lncRNA Carmn regulates the SRF/MYOCD complex in trans and promotes the expression of CArG box-dependent S-specific genes (e.g., MIR143/145, CNN1, TAGLN, TGFB11) to maintain the contractile phenotype of vascular smooth muscle cells. Thus lncRNA Carmn deletion leads to attenuated expression of SM-specific genes, which significantly impairs SMC contractility and gradually slows gastrointestinal motility, thereby inducing pseudo-obstruction leading to death [37]. Ovarian cancer is a heterogeneous gynecologic malignancy with many risk factors [38]. Serum expression of lncRNA FAM225B was reduced in ovarian cancer patients compared to healthy individuals. A decrease in proliferation, metastasis and invasion of ovarian cancer cells was found by restoring the expression of lncRNA FAM225B. RIP and CHIP experiments revealed the existence of a direct interaction between lncRNA FAM225B and DDX17 as well as binding between DDX17 and the PDIA4 promoter. DDX17 as a transcription factor, binds to the PDIA4 promoter to promote transcription, and restoration of PDIA1 expression inhibits the malignant phenotype of ovarian cancer cells. Therefore, in ovarian cancer, lncRNA FAM225B promotes PDIA4 expression through DDX17, which in turn inhibits ovarian cancer progression [39].

In contrast, LncRNAs can also inhibit the transcriptional activity of TFs by suppressing their binding to target genes (Fig. 1D). Y-Box binding protein 1 (YBX1) is an RNA-binding protein that can act as a transcription factor to regulate the transcription and expression of downstream genes. Under hypoxia, lncRNA HIF1A-AS3 directly binds to YBX1, inhibits its ability to bind to the p21 and AJAP1 promoters, and represses its transcriptional activity, thereby promoting progression of ovarian tumor [40].

Histone modification and DNA methylation are the main ways in which lncRNAs act on target genes through epigenetic inheritance, and this behavior mainly occurs in the nucleus. By recruiting histones and DNMT with different functions to the promoter regions of target genes, they directly promote or inhibit the transcription of the target genes, affecting the expression of the genes at the mRNA level, and playing a regulatory role in the development of diseases. Transcription factors bind to specific DNA sequences and also play a role in repressing or enhancing gene expression.

### 2.2 LncRNAs form a double feedback loop with target genes

**Fig. 2:**
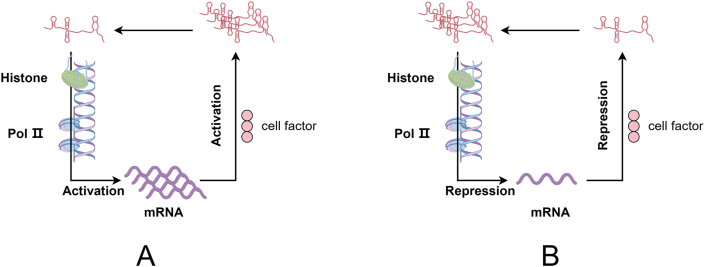
LncRNA forms a double feeder loop with the target gene. By Figdraw. **(A)** LncRNAs promote the transcription of target genes through epigenetic inheritance, while increased expression of target genes promotes the transcription of lncRNAs, and the two form a feedback loop that continuously promotes the transcription of target genes. **(B)** LncRNAs repress the transcription of target genes through epigenetic inheritance, and at the same time, the reduction of target gene expression will inhibit its inhibitory effect on lncRNAs, and both of them form a feedback loop to continuously inhibit the transcription of target genes.

The double feedback loop is a specific mechanism that exists on the basis of the epigenetic influence of lncRNAs on the transcriptional activity of target genes. This means that the lncRNAs positively regulates the transcription of the target gene while also being affected in turn by the target gene, thus forming a double feedback loop between the lncRNAs and the target gene (Fig. 2). The existence of the double feedback loop allows lncRNAs and genes to regulate each other to further enhance or attenuate the expression of the target gene. In the double feedback loop, when lncRNA promotes the transcription of a target gene, the target gene's regulation of the lncRNA tends to be promotive as well (Fig. 2A). Inflammatory bowel disease is a chronic autoimmune disease that occurs in the gastrointestinal tract, including ulcerative colitis and Crohn's disease, and its pathogenesis is similar to that of other autoimmune disorders, which have not yet been clarified [41]. The researcher found there is a double feedback loop between lncRNA CARINH and IRF1, in which lncRNA CARINH promotes the transcription of IRF1 through histones H3K27ac, while highly expressed IRF1 promotes the expression of lncRNA CARINH by directly binding to the lncRNA CARINH promoter, and thus the increased lncRNA CARINH through the feedback effect is able to further increase the expression level of IRF1. Follow-up studies revealed that gut microbes were able to maintain a double feedback loop of lncRNA CARINH and IRF1, while knockdown of lncRNA CARINH also altered the composition of gut microbes, suggesting that there is also a reciprocal relationship between this double feedback loop and gut microbes [42]. The expression of lncRNA HIF1A-AS2 was found to be up-regulated in NSCLC by transcriptomics. Further studies revealed that lncRNA HIF1A-AS2 could directly bind to DHX9 and recruit it to the MYC promoter to activate the transcription of MYC, while MYC was able to respond to the induction of KRAS and promote the expression of lncRNA HIF1A-AS2. LncRNA HIF1A-AS2 regulated and formed a double-feeding loop with MYC in both directions, which further enhanced the proliferation and tumor metastasis of lung cancer cells [43].

Double feedback loops can also provide sustained inhibition of target gene expression (Fig. 2B). Li [44] observed that SNAI2 could directly bind to the promoter region of lncRNA ELF3-AS1 and inhibit its transcription, and because ELF3 shares the promoter with lncRNA ELF3-AS1, SNAI2 could also inhibit the transcription of ELF3. Follow-up studies revealed that knockdown of lncRNA ELF3-AS1 decreased expression levels of miRNA targeting SNAI2, leading to upregulation of SNAI2 mRNA and protein expression, which activated the SNAI2 downstream signalling pathway as well as accelerated the cellular G1/S phase transition. SNAI2 and lncRNA ELF3-AS1 form a double negative feedback loop to maintain SNAI2 overexpression and continuously activate SNAI2 signaling while suppressing the expression of lncRNA ELF3-AS1 and ELF3. In turn the down-regulation of lncRNA ELF3-AS1 further activates SNAI2 signaling, which ultimately promotes cell proliferation and invasive metastasis, leading to a poor prognosis of GC.

The presence of double feedback loop allows the regulatory effect of lncRNA on downstream target genes to be further enhanced by feedback from target genes to the lncRNA, which is able to consistently activate or repress the transcription of the lncRNA and its downstream target genes. By reviewing relevant recent articles, we observed that the vast majority of double feedback loops belong to negative feedback loops, that is, the feedback effect of target genes on lncRNAs is consistent with the results of lncRNA regulation of target genes, and manifests itself as either enhancement or inhibition at the same time, which results in the further enhancement of the function of lncRNAs. The double feedback loop, because of the existence of a sustained action on the target gene, is a good explanation for disease progression as an ongoing process. It is easy to see from the three double feedback mechanisms mentioned in the article that lncRNAs play a role in promoting disease progression in both colitis and cancer, and that continued activation of signaling in the double feedback loop is one of the reasons for the continued deterioration of the disease, which adversely affects the patient.

### 2.3 LncRNAs are involved in alternative splicing of target genes

Alternative splicing (AS) is a process by which a single gene is eventually translated into multiple proteins. It is the process of rejoining RNAs in several ways during the process from precursor mRNA to mature RNA, resulting in different mRNAs that are translated into different proteins [45]. A study found the pre-mRNA of Mnk2 can be spliced into two isoforms, Mnk2a and Mnk2b, with Mnk2a containing the binding site for MAPK but not Mnk2b in GC. LINC00923 promotes the binding of hnRNPC to Mnk2 pre-mRNA at the e14a site, resulting that Mnk2 is spliced into more Mnk2b isoforms. As a result, the expression of Mnk2a with MAPK binding site was significantly decreased, so the activation of MAPK/PPARα signaling pathway received inhibition, which ultimately promoted the development of peritoneal metastasis of GC [46]. SRSF6 is a classical splicing factor that has now been found to play an important role in colorectal cancer (CRC) and an unknown role in GC [47]. PICALM has two subtypes PICALML and PICALMS, PICALML has twenty-two exons that enhance oxaliplatin resistance, whereas PICALMS lacks exon 14 compared to LICALML, resulting in chemosensitivity in GC. The researchers found that lncRNA CRNDE was able to induce ubiquitination of SRSF6 and promoted its degradation by the ubiquitin-proteasome pathway. When SRSF6 is degraded, the isoform of PICALM is shifted from the l-type to the s-type, and the reduction of PICALML, which has more drug-resistant exons, leads to the attenuation of oxaliplatin resistance. Thus, overexpression of lncRNA CRNDE attenuates resistance to oxaliplatin in GC patients and improves the therapeutic efficiency of patients by decreasing the stability of SRSF6, which results in a decrease in l-type expression during SRSF6 splicing of PICALM [48].

**Fig. 3:**
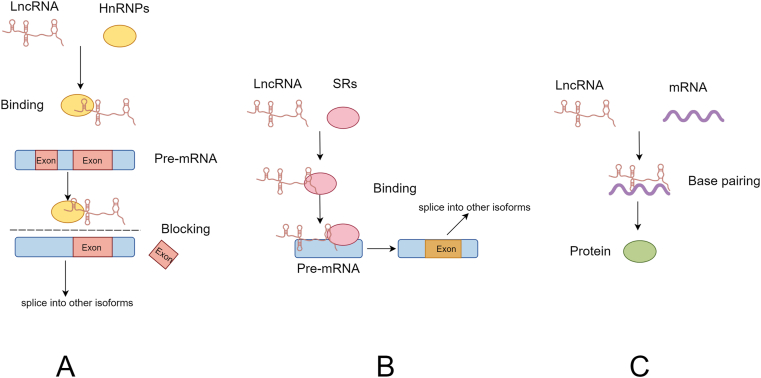
LncRNAs are involved in post-transcriptional modification of target mRNAs. LncRNAs induce transformation of target mRNAs into other isoforms by alternative splicing, with the help of HnRNPs **(A)** or SRs **(B)**. **(C)** LncRNAs enhance or inhibit the translation of mRNAs by directly base-pairing with target mRNAs.

The heterogeneous nuclear ribonucleoprotein (hnRNP) family and the serine/arginine-rich splicing factor (SR) family are two families that are closely related to AS. Therefore, we can classify the involvement of lncRNAs in AS into two ways, i.e., lncRNAs regulate the AS process of mRNAs by resorting to hnRNPs or SRs, thereby inducing the translation of mRNAs into different protein isoforms(Fig. 3A and B). The occurrence of AS does not generally increase or decrease the expression of the target gene, but rather results in its pre-mRNA being sheared into different isoforms, which leads to changes in the function of the target gene.

### 2.4 LncRNAs interact directly with mRNAs through base pairing

Before mRNAs begin translation, lncRNAs can directly interact with mRNAs via base pairing to enhance their translation (Fig. 3C). LncRNA Uchl1-AS is present on the reverse strand of the Uchl1 gene and enriched in the nucleus. Carrieri found that upon rapamycin treatment, lncRNA Uchl1-AS shuttles from the nucleus to the cytoplasm, where it targets the Uchl1 mRNA and increases Uchl1 translation through its embedded inverted SINEB2 sequence [49]. Identically, lncRNA NR4A1AS is present on the reverse strand of the NR4A1 gene and is positively correlated with NR4A1 mRNA expression. By constructing different deletion transcripts of lncRNA NR4A1AS, it was found that lncRNA NR4A1AS stabilized NR4A1 mRNA by forming an RNA-RNA complex with NR4A1 mRNA via partial base-pairing, which ultimately up-regulated NR4A1 expression in CRC cells [50].

However, this base-pairing behavior also has the potential to inhibit mRNA translation. Lnc-SMaRT is a key regulator during skeletal muscle differentiation in mice. The translation of Spire1 mRNA was found to be regulated by the lnc-SMaRT/DHX36 molecular complex. The role of DHX36 which acts as an RNA helicase is to solve the G-quadruplex structure on Spire1 mRNA, allowing its base pairing with lnc-SMaRT. The lnc-SMaRT base-pairs with the G4 region of Spire1 mRNA immediately after the resolving activity of DHX36 has occurred, thus inhibiting subsequent G4 refolding which ultimately inhibits the translation of Spire1 mRNA [51].

Similar regulatory mechanisms exist in many tumor diseases. In HCC, lncRNA ICR upregulates ICAM-1 expression by increasing the stability of its mRNA through RNA duplex formation [52]; in GC, lncRNA MACC1-AS1 promotes the stability of MACC1 mRNA by physically binding to MACC1 mRNA [53]; In glioma, lncRNA PTB-AS could directly bind to the 3′ UTR region of PTBP1 mRNA, which masks the binding site of miR-9 in the PTBP1, resulting in the inability of miR-9 to recognize PTBP1, thus enhancing the stability of PTBP1 [54]. These studies suggest that lncRNAs can enhance mRNA stability and facilitate mRNA translation by forming stable RNA-RNA complexes through direct base pairing with their mRNA targets. This behavior belongs to a type of post-transcriptional modification of lncRNAs involved in target genes and occurs mainly in the cytoplasm.

## 3 Direct interaction of lncRNAs with proteins

### 3.1 Involved in post-translational modification of target proteins

Protein post-translational modification occurs in some proteins that are inactive after translation and still need to go through a series of processing and modification before they can become functionally mature proteins. The process of post-translational modification is reversible and consists mainly of ubiquitination, phosphorylation, acetylation and glycosylation and lncRNA is extensively involved in these processes [55].

#### 3.1.1 Ubiquitination

**Fig. 4:**
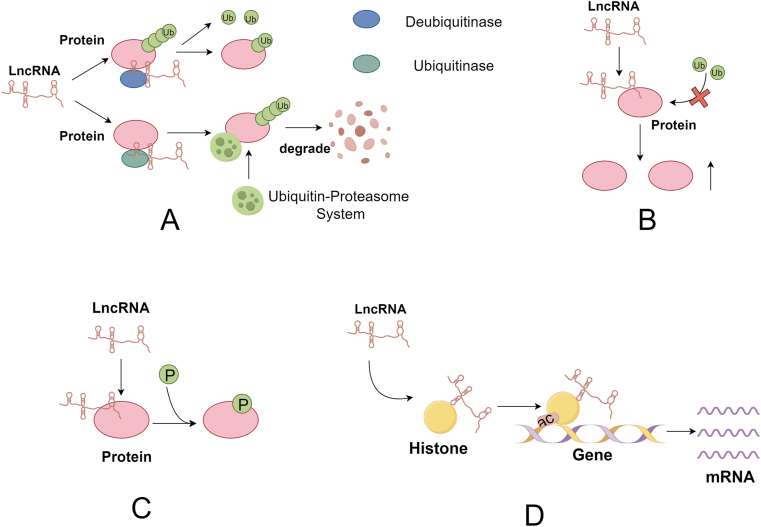
LncRNAs are involved in post-translational modification of target proteins.By Figdraw. **(A)** LncRNAs promote ubiquitination (or deubiquitination) of target proteins by recruiting ubiquitinase (or deubiquitinase) to them. **(B)** LncRNAs avoid ubiquitination of target proteins by binding to and occupying their ubiquitination sites. **(C)** LncRNA can promote phosphorylation of target proteins. **(D)** lncRNA promotes histone acetylation and thus downstream gene transcription.

Ubiquitination is the process by which ubiquitin is covalently bound to a target protein by the catalytic action of various ubiquitinases and thus degraded by the proteasome. The process of ubiquitination requires the synergistic action of ubiquitin-activating enzymes, ubiquitin-binding enzymes and ubiquitin ligases [56]. LncRNAs are directly involved in the ubiquitination process of target proteins through ubiquitinases or deubiquitinases (Fig. 4A). LINC00240 is transcribed from the 6p22.1 GC risk locus and is highly expressed in GC tissues. Highly expressed LINC00240 promotes the progression of GC, and its role in promoting the proliferation and metastasis of GC cells has been observed both in vivo and in vitro. Mechanistically, LINC00240 enhances the binding of DDX21 to its novel deubiquitinating enzyme USP21, leading to deubiquitination of DDX21 and avoiding its degradation by the ubiquitin-proteasome system, which enhances the stability of DDX21 and promotes the progression of GC [57]. SNHG11 is an autophagy-related lncRNA that is highly expressed in GC patients and promotes GC progression. Silencing of lncRNA SNHG11 revealed no change in GSK-3β expression at the mRNA level, instead upregulation at the protein level. RIP experiments showed that lncRNA SNHG11 interacts with the ubiquitin ligase CUL4A and promotes ubiquitination of GSK-3β. GSK-3β is an inhibitor of the Wnt/β-Catenin pathway. So lncRNA SNHG11 degradated the GSK-3β protein and thus attenuates its inhibitory effect on the Wnt/β-Catenin pathway, and ultimately promotes GC progression [58].

LncRNAs can not only directly ubiquitinate target proteins through ubiquitinases, but also indirectly inhibit the ubiquitination of target proteins by disrupting the recognition between ubiquitinases and target proteins (Fig. 4B). Glioblastoma multiforme (GBM) is considered the most aggressive primary brain tumor in adults [59]. During the treatment of GBM patients, the lncRNA PDIA3P1 was found to be more highly expressed in temozolomide-resistant patients. MDM2 is an E3 ubiquitin ligase. LncRNA PDIA3P1 could stabilize C/EBPβ protein by disrupting the C/EBPβ- MDM2 complex and avoiding ubiquitination of C/EBPβ protein by MDM2, which reduces the sensitivity of glioma cells to temozolomide treatment [60].

The ubiquitin-proteasome system is a very common mode of protein degradation, in which proteins are first modified by ubiquitination and then degraded by the proteasome system. LncRNAs are able to influence the ubiquitination of downstream target proteins through various ubiquitinating enzymes as well as deubiquitinating enzymes, and ubiquitinyl-modified target proteins are more likely to be degraded by the proteasome. Therefore, the ubiquitin molecules of target proteins are removed by deubiquitinating enzymes, which enhances the stability of the target proteins and enables the target proteins to fully perform their functions. On the contrary, after ubiquitination of the target protein is induced by ubiquitinating enzymes, the target protein with added ubiquitin molecules is degraded, which reduces the stability of the target protein and inhibits the function of the target protein.

#### 3.1.2 Phosphorylation

Phosphorylation is the addition of a phosphorylation group to the amino acid residues of a target protein, thereby activating the potential of the target protein [61]. LncRNAs can participate in the phosphorylation process of target proteins and thus affect the activity of them (Fig. 4C). The activation of YAP is critical for several cancers, including CRC, and can drive tumorigenesis and progression. LncRNA GAS5 showed negative correlation with YAP expression in several CRC cell lines, and YAP protein accumulated when GAS5 was inhibited. Subsequent experiments demonstrated that GAS5 was able to directly interact with the WW structural domain of YAP promoting the release of YAP from the nucleus to the cytoplasm as well as the level of YAP phosphorylation at serine 127. Phosphorylation of YAP promotes its ubiquitination process, making it more susceptible to degradation by the proteasome system，leading to a decrease in YAP expression. Thus lncRNA GAS5 inhibits YAP signaling by promoting YAP phosphorylation, which promotes its ubiquitination and degradation, thereby inhibiting CRC progression [62]. LncRNA LIST was highly expressed in NSCLC tissues and the expression level of LIST was significantly correlated with TNM stage. The Y530 site of c‐Src is located in the SH1–C domain, where LIST fragment 6 binds. Immunoprecipitation and mass spectrometry assay showed that knockdown of LIST significantly enhanced the interaction between protein kinases (CHK and CSK) and c-Src, and CHK and CSK were able to promote the phosphorylation of Y530. Meanwhile，c-Src can positively regulate LIST transcription by activating the NF-κB signaling pathway and recruiting the P65 transcription factor into the LIST promoter, thus forming a positive feedback pathway between LIST and c-Src that further promote tumor progression and drug resistance [63]. LncRNA ZNF593-AS directly interacts with the truncation-deleting signal response domain of interferon regulatory factor 3 (IRF3)，inhibits fatty acid-induced phosphorylation of IRF3，and reduces IRF3 activity, which contributes to the amelioration of cardiomyocyte cell death and body inflammation [64]. The interaction of SMG5 and PP2A is required for UPF1 to be dephosphorylated in an evolutionarily conserved manner. LncRNA DDIT4-as1 promotes the phosphorylation of UPF1 by preventing the binding of SMG5 and PP2A to UPF1. Activation of UPF1 phosphorylation decreases DDIT4 mRNA stability and activates the mTOR pathway, leading to resistance to gemcitabine therapy [65].

Ubiquitination and phosphorylation are two different processes. Ubiquitinated proteins are more susceptible to degradation by the proteasome system, which reduces the stability of proteins; whereas phosphorylation aims to enhance the activity of proteins through the addition of phosphorylated groups, stimulating the proteins to be able to perform their functions more fully. The process of phosphorylation is also reversible, and protein phosphorylation is closely related to cancer development and treatment. In treatment, we can regulate the ratio of phosphorylated proteins to make the cancer develop in a more favorable direction, so as to achieve the goal of cure.

#### 3.1.3 Acetylation

Acetylation is the process of adding an acetyl group to a protein lysine residue or N-terminus catalyzed by an acetyltransferase enzyme, which plays an important role in the regulation of protein function, chromatin structure, and gene expression [66]. LncRNA EPB41L4A-AS1 significantly affects not only cancer cell energy metabolism but also mediates glucose metabolism in diabetes mellitus, which is highly expressed in liver and muscle tissues of diabetic patients. PGC1-β is a transcriptional coactivator whose activity is inhibited by GCN5/KAT2A-mediated lysine acetylation. Overexpression of lncRNA EPB41L4A-AS1 promotes the acetylation of histone H3K27 and PGC1-β in the promoter region of GLUT4 by interacting with GCN5, leading to loss of transcriptional facilitation, leading to inhibit GLUT4 gene expression, and cellular uptake of glucose [67].

Histones are the main substrates of acetyltransferases, and acetylation of histones affects gene expression. Thus lncRNAs regulate target gene expression by modulating histone acetylation (Fig. 4D). LncRNA PVT1 expression is upregulated in nasopharyngeal carcinoma patients and is associated with low patient survival. lncRNA PVT1 interacts with KAT2A acetyltransferase to promote the acetylation of histone H3K9 and its recruitment to the NF90 promoter region, which stabilizes the expression of HIF-1α and ultimately leads to tumor cell proliferation [68].

Early acetylation studies focused on histones, whose acetylation and deacetylation are respectively mediated by histone acetyltransferases (HATs) and histone deacetylases. Acetylation of histones neutralizes the positive charge of lysine thereby facilitating the relaxation of chromosomal structure, which leads to the binding of various transcription factors to DNA and promotes gene transcription [69]. HATs can be categorized into three families: the GNAT family (including KAT2A, ELP3, etc.), the MYST family (including MOZ, TIP60, etc.), and the orphan family (including p300, etc.) [70]. The regulation of histone acetylation by LncRNAs is mediated by a variety of HATs, and members of all three families are involved. LncRNA TMPO-AS1 promotes the acetylation of H3K27 through p300 [71]; LINC00839 promotes the acetylation of H4K5 and H4K8 through Tip60 [72]; lncRNA AFAP1-AS1 interacts with KAT2B to promote acetyltransferase activity and thus promote the acetylation of H3K14 [73]. LncRNAs regulate the transcription of many different functional genes through histone acetylation, and therefore produce a variety of effects. And there have been numerous experiments demonstrating that lncRNAs are closely related to tumour cell proliferation and metastasis, macrophage polarization, and apoptosis during cellular senescence under this mechanism [[74], [75], [76]].

### 3.2 Enhancement the stability of target proteins

**Fig. 5:**
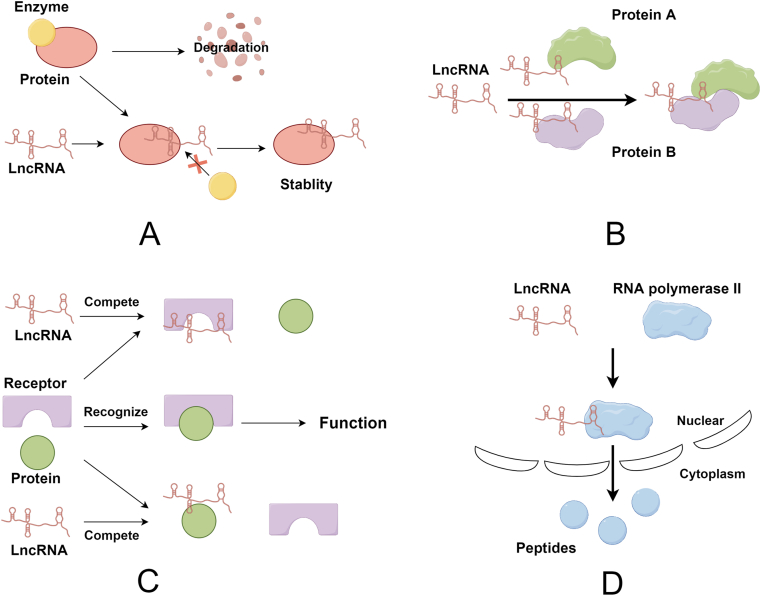
LncRNAs regulate target proteins in other forms.By Figdraw. **(A)** LncRNAs promote target protein stability by inhibiting the binding of target proteins to enzymes to avoid target protein degradation. **(B)** LncRNAs act as brackets to recruit two (or more) proteins together and facilitate recognition between proteins. **(C)** LncRNAs occupy protein-receptor binding sites located on target proteins (or target protein receptors) and inhibit the target proteins from performing their functions. **(D)** LncRNAs are capable of encoding peptides.

The participation of LncRNA in post-translational modification of target proteins actually belongs to a kind of regulation of the amount of target protein expression. In addition, lncRNAs can also promote the expression of target proteins by enhancing the stability of target proteins. Such lncRNAs fail to directly regulate the amount of target protein by participating in the transcription and translation process of the target protein, but indirectly regulate the expression of the target protein through the formation of a stable complex between the two, which makes the target protein less susceptible to degradation by enzymes and other substances and enhances its stability (Fig. 5A). The involvement of lncRNAs in the ubiquitination of target proteins mentioned above also belongs to the regulation of target protein stability to some extent. In GBM, lncRNA LINREP interacts with PTBP1 and HuR protein complex to protect PTBP1 from ubiquitin-proteasomal degradation, which in turn is involved in AS regulation mediated by PTBP1 [77]. Some scholars studying human aging have found that down-regulation of lncRNA MAGI2-AS3 delayed cellular aging. LncRNA MAGI2-AS3 was able to promote the degradation of HSPA8 through the proteasome pathway and reduce the stability of HSPA8 protein. Therefore, low expression of lncRNA MAGI2-AS3 can inhibit the degradation of HSPA8 by the proteasome, which reduces the hydrogen peroxide level and stabilizes the HSPA8 protein level [78]. Dysregulated secretion of very low-density lipoprotein (VLDL) in the liver leads to nonalcoholic fatty liver disease, and the expression of lncRNA RHL was found to be significantly higher in patients when they were treated with a high-fat diet. It was found that lncRNA RHL enhances the stability of heterogeneous nuclear ribonuclear protein U (hnRNPU) by directly binding, and hnRNPU can transcriptionally activate Bmal1, thus inhibiting the secretion of VLDL in stem cells and maintaining lipid homeostasis in the body [79].

### 3.3 Role of brackets

LncRNAs act as scaffolds capable of bringing together multiple proteins to form complexes, which facilitate the binding of proteins to each other and participate in various physiological processes in the human body (Fig. 5B). The binding of PD-L1 on tumor cells to PD-1 on activated T cells can promote immune evasion of tumor cells, so how to reduce the binding of PD-L1 to PD-1 has become one of the keys to tumor therapy [80]. It was found that lncRNA HITT was *trans*-activated under the stimulation of IFN-γ, which led to the reduction of PD-L1 translation, as well as inhibition of immune escape from tumors, by binding to Regulator of G Protein Signalling 2 (RGS2) and coordinating the binding of RGS2 to the 5′ UTR of PD-L1 [81]. LncRNA AC09894.1 can act as a scaffold molecule that recruits USP3 to the androgen receptor (AR), promotes USP3-mediated deubiquitination of the AR, and improves the stability of the AR. AR as a transcription factor promotes the transcription of RASGRP3, and the increased expression of RASGRP3 ultimately facilitates the apoptosis of CRC cells by activating the MAPK signaling pathway [82]. LncRNA CACClnc plays an important role in CRC chemoresistance, and it was found that lncRNA CACClnc specifically binds to YB1 and U2AF65 (a subunit of the U2AF splicing factor) and promotes the YB1 and U2AF65 interaction, which in turn regulates the AS process of RAD51 mRNA [83]. LncRNA Smyca is also one of the targets for overcoming tumor chemoresistance, and lncRNA Smyca enhances the TGF-β/Smad signaling pathway by acting as a scaffold that promotes Smad3/Smad4 binding, and further serves as a Smad target to amplify the TGF-β signaling pathway thereby promoting tumor progression [84].

### 3.4 Alternative ceRNA: inhibition of target protein binding to downstream targets

LncRNA as ceRNA can not only bind to miRNA to play its role, but also can avoid the binding of target proteins to the downstream sites through competitive action, which in turn can inhibit the function of target proteins without changing the number and outcome of target proteins (Fig. 5C). LncRNA GLTC acts as a binding partner of LDHA to inhibit the regulatory effect of SIRT5 on LDHA by competitively binding LDHA with SIRT5, promoting the succinylation of LDHA at the lysine 155 site, thereby promoting the enzymatic activity of LDHA, and thus promoting the progression of thyroid cancer [85]. In TNBC, lncRNA TGFB2- AS1 was found to be able to competitively bind SMARCA4 (the core subunit of the SWI/SNF complex) with TGFB2, preventing the complex from binding to the promoter of TGFB2. Thus overexpression of lncRNA TGFB2- AS1 significantly inhibited the enrichment of SMARCA4 in the TGFB2 promoter region, which in turn inhibited TGFB2 transcription and suppressed tumor growth and lung metastasis [86]. LncRNA HOXC13-AS has the ability to regulate keratinocyte differentiation and its expression rises gradually during epidermal wound healing. It was found that COPA protein is a part of COATOMER protein complex I (COPI), and the ability of LncRNA HOXC13-AS to capture COPA protein leads to the inability of the COPI complex to function due to the lack of COPA, which results in the inability of COPI to retrogradely transport cargo proteins from the Golgi to the endoplasmic reticulum, leading to endoplasmic reticulum stress and promoting keratinocyte differentiation [87].

The competitive role of LncRNA is reflected in two aspects: one is that lncRNA binds to downstream targets, reducing the number of targets that can be recognized and bound by target proteins and thus inhibiting the target proteins from binding downstream targets. The other is that lncRNA binds to the target proteins and reduces the number of target proteins that can participate in the binding of downstream targets, thus inhibiting the target proteins from binding downstream targets.

## 4 Encoded peptides

LncRNA, as a type of ncRNA, was initially thought to be incapable of coding proteins, but scholars have found that lncRNA is also transcribed by RNA polymerase II and has a structure similar to that of mRNA, which suggests that lncRNA may also have the ability to translate proteins (Fig. 5D) [88]. With the development of ribosome sequencing technology, it has been found that some lncRNAs have the ability to bind to ribosomes, and the sORFs in certain lncRNAs may be one of the sources of proteins, suggesting that these lncRNAs may have the ability to encode polypeptides [89]. CVD is one of the most dangerous diseases in the world today. The pathological hallmark of CVD development is the change of vascular smooth muscle cells from contractile to proliferative [90]. LncRNA PSR was found to be significantly up-regulated during vascular remodeling. Although lncRNA PSR belongs to a kind of lncRNA, it was demonstrated that it also encodes a small peptide, which was named arteridin. The transcription of arteridin is regulated by lncRNA PSR, and the transcription of lncRNA PSR is also induced by arteridin, so the two regulate each other, forming a feedback loop, which further affects the downstream genes through the transcription factor YBX1, thus exacerbating the process of vascular remodeling, and adversely affecting CVD [91]. When inflammation occur, lncRNA MIR155HG is highly expressed in antigen-presenting cells, and lncRNA MIR155HG encodes a 17-amino-acid polypeptide named p155. p155 binds to adenosine of HSC70, which is required for antigen presentation by DC cells, and prevents antigen presentation by DC cells as well as T cell activation [92]. LINC00961 encodes SPAAR in endothelial cells and its expression increases during differentiation. LINCC00961 deletion significantly reduces endothelial cell proliferation, migration, and barrier integrity [93]. Taking everything together, these studies highlight the fact that lncRNAs have the function of encoding peptides and that these peptides play important roles in both physiological and pathological processes in the human body (Table 1).

**Table 1:** lncRNAs capable of encoding polypeptides and their functions.

LncRNA	Peptide	Amino acid number	Functionality	References
LncRNA MIR155HG	P155	17	Preventing antigen presentation by DC cells and T cell activation	[92]
LINC00961	SPAAR		Promotes angiogenesis	[93]
HOXB-AS3	HOXB-AS3 peptide	53	Inhibits the growth of CRC	[94]
LINC00467	ASAP	94	Promotes proliferation of CRC cells	[95]
LINC00665	LINC00665_18aa	18	Inhibition of tumor cell viability, proliferation and migration	[96]
LINC00511	LINC00511-133aa	130	Promotes invasiveness and stemness of breast cancer cells and apoptosis	[97]
MIR7-3	MIR7-3HG-ORF	125	Relief of pancreatic β cell dysfunction due to the use of dexamethasone.	[98]

## 5 Summary

The research of lncRNA mechanism is a gradual process. At the beginning, lncRNAs did not receive much attention due to their non-coding properties, but the discovery of the ceRNA mechanism made researchers realize that lncRNAs play an important role in the development and treatment of various diseases, so lncRNAs have become a hot topic in the academic research field at present. With years of research, it has been found that other mechanisms of lncRNAs are diverse. At the transcription level, it can act on the promoter of target genes and promote the transcription of target genes through epigenetic inheritance, and change the structure of the protein encoded by target genes through variable shearing; at the translation level, it can stabilize the structure of target proteins by directly binding to the binding domains of target proteins so as to avoid their degradation, influence post-translational modification of target proteins, and act as scaffolding proteins to attract a variety of proteins, and so on. LncRNA research is constantly updating the understanding, and in recent years with the development of ribosome sequencing technology researchers have discovered that some lncRNAs also have the ability to encode and influence disease progression by coding for polypeptides that are low in amino acids. LncRNA also has a place in immunology, which acts on immune cells and influences their release of inflammatory factors through various pathways, and is able to promote immune cell proliferation and activation, but of course there also are lncRNAs that promote apoptosis of immune cells and inhibit immune cell activation.

In the clinic, lncRNA can be used as a molecular marker to predict patient prognosis. Li [99] predicted the prognosis of patients with Uterine Corpus Endometrial Carcinoma by constructing a potential prognostic model consisting of 14 disulfidptosis associated lncRNA prognostic markers. Cheng [100] used a machine learning algorithm to construct a novel lncRNA signature associated with cuproptosis to predict prostate cancer prognosis. Disease prognostic models constructed from lncRNAs may help to improve precision treatment of patients and improve their clinical outcomes. LncRNAs can also be used as diagnostic markers, and a number of assay kits exist to facilitate the diagnosis of diseases aided by the detection of data characterising lncRNAs in patients' body fluids [[101], [102], [103]].

Although lncRNAs have been implicated in the development of many diseases, they are currently only used as a new biomarker to help one predict patient prognosis or to aid in the diagnosis of disease. How to apply lncRNA to disease treatment remains a challenge. Continued in-depth research on the mechanism of lncRNA will provide us with new ideas as well as new tools for the treatment of diseases in the future.

## CRediT authorship contribution statement

**Jiahao Zhang:** Writing – review & editing, Writing – original draft. **Huike Zhu:** Writing – review & editing. **Linjing Li:** Writing – review & editing. **Yuting Gao:** Writing – review & editing. **Boyi Yu:** Writing – review & editing. **Guorong Ma:** Writing – review & editing. **Xiaodong Jin:** Writing – review & editing, Supervision. **Yingbiao Sun:** Supervision.

## Declaration of competing interest

The authors declare no competing interests.
